# Fabrication of Buried Microchannels with Almost Circular Cross-Section Using HNA Wet Etching

**DOI:** 10.3390/mi15101230

**Published:** 2024-09-30

**Authors:** Qihui Yu, Henk-Willem Veltkamp, Remco J. Wiegerink, Joost C. Lötters

**Affiliations:** 1 MESA^+^ Institute for Nanotechnology, University of Twente, 7522 NB Enschede, The Netherlands; h.veltkamp@utwente.nl (H.-W.V.); r.j.wiegerink@utwente.nl (R.J.W.); j.c.lotters@utwente.nl (J.C.L.); 2 Institute for Biomedical Engineering, University and ETH Zürich, 8092 Zürich, Switzerland; 3 Paul Scherrer Institut (PSI), 5232 Villigen, Switzerland

**Keywords:** buried channel technology, HNA etching, microfluidic channel, Coriolis mass-flow sensor, 81.65.Cf, 81.15.-z, 81.15.Gh, 52.77.-j

## Abstract

In this paper, a novel fabrication process for the realization of large, suspended microfluidic channels is presented. The method is based on Buried Channel Technology and uses a mixture of HF, HNO_3_, and water etchant, which has high selectivity between the silicon substrate and the silicon-rich silicon nitride mask material. Metal electrodes for actuation and read-out are integrated into the fabrication process. The microfluidic channels are released from the silicon substrate to allow the vibrational movement needed for the application. The resulting microfluidic channels have a near-circular cross-section, with a diameter up to 300 μm and a channel wall thickness of 1.5 μm. The structure of a micro-Coriolis mass-flow and density sensor is fabricated with this process as an example of a possible application.

## 1. Introduction

In 2007, Dijkstra et al. proposed so-called Surface Channel Technology (SCT), which allows the fabrication of suspended tubes with a thin silicon-rich silicon nitride (SiRN) tube wall in a silicon substrate [1]. In this process, the channel is etched in a silicon substrate through small slit openings in a mask layer, and the slits are subsequently closed during the channel wall deposition by low-pressure chemical vapor deposition (LPCVD). At the end of the process, the channel is released from the silicon substrate using a (semi-)isotropic etch step. The resulting flow tubes usually have a semicircular cross-sectional area with a thin flat top side. Based on this fabrication process, Haneveld et al. demonstrated a micro-Coriolis mass-flow sensor [2]. In the next decades, many studies of micro-Coriolis mass-flow sensors fabricated with SCT were carried out [3,4,5,6,7,8].

However, the cross-sectional dimensions of the channels fabricated with SCT are limited, usually with a width from 40–80 μm [9], which then limits the flow range through the channel because of the resulting pressure drop. Moreover, the flat top membrane will deform under the pressure of the fluid in the channel, which causes a change in channel geometry and mechanical properties (for example, stiffness, resonance frequency, etc.). Although this feature can be used for the integration of a pressure sensor by adding strain gauges on top of the membrane [10], it also introduces a pressure dependence when the channels are used in a mass flow sensor. Therefore, new fabrication methods need to be explored and investigated in order to realize microfluidic channels with large and circular cross-sections while keeping the advantages of the SCT channel (i.e., thin and chemically inert channel walls, easy integration of actuation/readout electrodes, etc.).

Buried Channel Technology (BCT) was first proposed in the late 1990’s [11,12]. This technology makes it possible to fabricate microfluidic channel structures with an almost circular cross-sectional area in a silicon substrate with a single photolithographic mask. Several works related to this technology were published in the following decades [13,14,15]. However, the fabricated channels in these works are usually less than 100 μm in diameter. In 2019, Groenesteijn et al. [16] presented the fabrication of channels by using BCT and xenon difluoride (XeF_2_) etch. Channel widths up to 160 μm were achieved with a channel wall thickness of 5 μm, resulting in a diameter-to-wall thickness ratio of 32. Due to the change in etching kinetics of XeF_2_ when the etched structure becomes larger than 120 μm, the shape of the cross-section turns more hexagonal. In 2021, a microfluidic channel with a circular cross-section was successfully fabricated based with BCT [17]. The cross-section has a diameter of 100 μm. The etching method used to etch the bulk silicon material is wet isotropic silicon etching, also known as HNA etching, which uses a mixture of HF, HNO_3_ and water [18,19,20,21,22]. Although the fabricated channel was not closed and not released from the silicon substrate, still, a great potential for realizing a microfluidic channel with circular and large cross-sections is shown.

In the present work, a complete fabrication process based on BCT with modified steps from a bare silicon substrate to a suspended microfluidic channel is presented. A fabrication run is carried out to demonstrate this process. The process is designed to achieve a channel diameter of 300 μm and a single layer SiRN channel wall of only 1.5 μm thick. The resulting channel diameter to wall thickness ratio is 200, while the highest reported value in literature is 75 [23]. To demonstrate the technology, the structure of a Coriolis mass-flow and density sensor is fabricated, which includes the required metal electrodes for actuation and sensing. This paper is organized as follows. First, in Section 2, an overview of the process is presented. A detailed description is given in Section 3, followed by a discussion in Section 4.

## 2. Process Design

**Figure 1:**
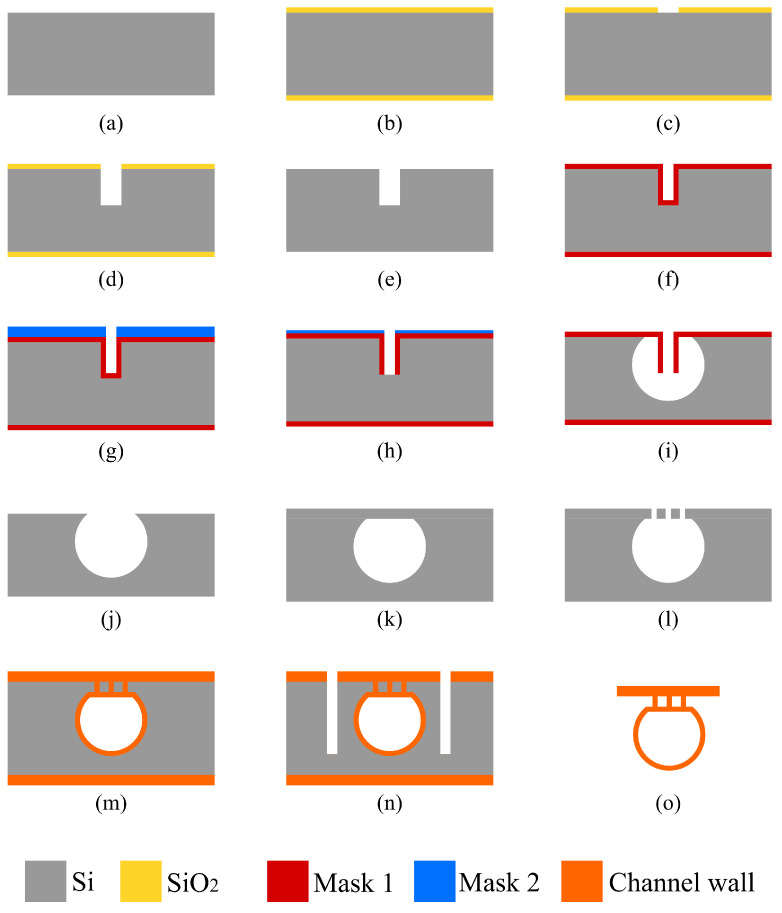
Overview of fabrication steps of modified BCT with HNA etching and wafer bonding: (**a**) Bare silicon wafer. (**b**) Thermal oxidation. (**c**) Patterning oxide layer. (**d**) Bosch-based trench etch. (**e**) Stripping of oxide layer. (**f**) LPCVD mask 1. (**g**) PECVD mask 2. (**h**) Trench bottom coating layer etch. (**i**) HNA channel etching. (**j**) Stripping of mask 1. (**k**) Silicon membrane bonding. (**l**) Patterning silicon membrane. (**m**) LPCVD channel wall. (**n**) Trench etching. (**o**) Channel release etching.

An overview of the fabrication process presented in this work for a sealed microfluidic channel is illustrated in Figure 1. The process is based on Buried Channel Technology (BCT) [11,16], but some key steps are modified and added to allow for the large channel diameter over wall thickness ratio. First, the silicon substrate is oxidized (step (b)) and this oxide layer is patterned (step (c)). This layer serves as a hard mask for etching a trench in the silicon substrate (step (d)), after which the oxide layer is stripped (step (e)). A coating layer is deposited by LPCVD (step (f)) followed by another coating layer by plasma-enhanced chemical vapor deposition (PECVD) (step (g)). The trench bottom is etched directionally and opened while the substrate surface is protected by the PECVD layer (step (h)). Silicon is then etched isotropically through the trench bottom opening using an HNA solution and the microfluidic channel structure is created (step (i)). The remaining mask layer is stripped (step (j)), and the channel is closed by bonding a silicon membrane on top (step (k)). This membrane is etched (step (l)) to gain access to the channel for the channel wall deposition step. The channel wall is deposited by LPCVD (step (m)). Due to the large cross-sectional area of the channel, two trenches are etched parallel to the microfluidic channel (step (n)) to shorten the time of the final release etch. At the end, the bulk silicon around the microfluidic channel is removed to suspend the channel, making it capable to vibrate (step (o)).

**Figure 2:**
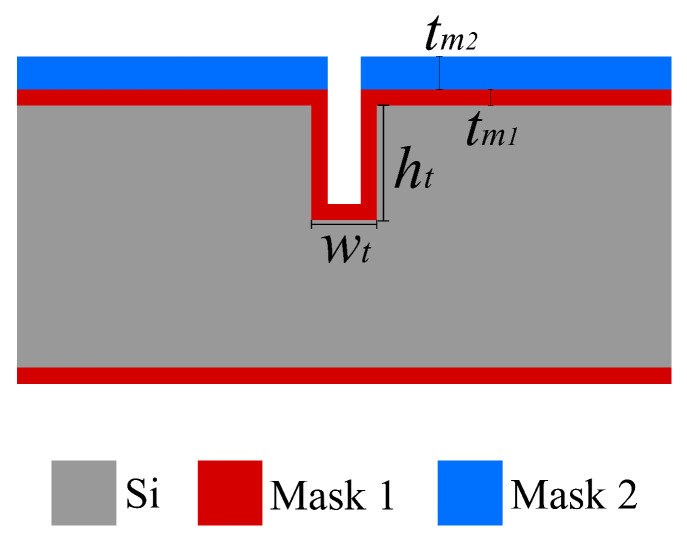
Important parameters in the trench bottom coating layer etch step: $w_{t}$ is the trench width, $h_{t}$ is the trench depth, $t_{m1}$ is the thickness of mask 1, and $t_{m2}$ is the thickness of mask 2.

In this process, the key steps are step (h), the trench bottom removal etching; step (i), the channel etching; and step (m), the channel wall formation, as illustrated in Figure 1. Figure 2 illustrates a schematic cross-sectional view of the trench structure before the bottom coating layer removal step with the relevant parameters (trench depth $h_{t}$, trench width $w_{t}$, channel etch mask thickness $t_{m1}$, and bottom coating layer etch mask thickness $t_{m2}$).

### 2.1. Trench Bottom Removal

For the trench bottom coating layer etch (step (h)), all listed parameters play important roles. In this step, the goal is to etch the trench bottom coating layer (mask 1) completely while the surface coating layer (mask 2) should not thin down. The bottom coating layer is harder to be etched if the trench is deeper ($h_{t}$↑) or narrower ($w_{t}$↓), i.e., having a larger aspect ratio. The total etching time required is proportional to the thickness of deposited layer $t_{m1}$, and $t_{m2}$ determines the maximum allowed etching time. Therefore, the optimal condition for this step is that the trench is shallow and wide, mask 1 is thin, and mask 2 is thicker.

### 2.2. Channel Etching

For the channel etch step (step (i)), a deeper or narrower trench (higher aspect ratio) will result in a much slower etch rate since the etchant needs to go through it to reach the silicon etchable surface. Thicker mask layers can help in this step as it allows longer etching times. The optimal condition for this step is that the trench is shallow and wide while mask 1 is thick. For the etched channel dimension, two parameters are decisive. The trench depth defines the maximum diameter of the channel while the mask layer thickness defines the maximum allowed etching time. To realize a large channel, a deeper trench and thicker mask layer are then necessary.

### 2.3. Channel Wall Formation

The channel wall (step (m)) formation is performed by LPCVD through the openings in the bonded membrane. LPCVD achieves conformal deposition because it relies on gas-phase reactions at reduced pressures, which allows the precursor gases to diffuse uniformly into complex surface features. The lower pressure enhances the mean free path of gas molecules, promoting uniform distribution and surface reaction. This ensures that the deposition occurs evenly on all surfaces, including vertical walls and deep trenches. The thickness of the channel wall is limited by the width of these openings and the maximum allowed deposited layer thickness is defined by layer properties (e.g., stress) and/or machine limitations. Therefore, the parameters of the etched trench do not have a direct effect on this step, but it does affect the design of the pattern of the opening. Moreover, the mechanical strength of the bonded membrane should also be considered, which is directly determined by the width of the trench.

### 2.4. Summary

To summarize, it can be seen that for different steps, the effect of the same parameters can be totally opposite. Table 1 summarizes these considerations. For instance, a deeper trench is preferred in realizing a large channel but not in the bottom removal step. Therefore, trade-offs must always be considered. The effects of these parameters are correlated themselves as well. For instance, a thicker mask 1 layer can allow longer channel etch time but it also narrows the trench, which can have a negative effect on the channel etch step. Moreover, in some extreme cases, for instance, if the trench is narrow enough, the thickness of the deposited mask layers cannot exceed a certain value; otherwise, the trench is filled.

## 3. Fabrication Process

In this section, the proposed fabrication process is discussed in detail. It is divided into different parts and described with details and discussions of the key steps. Table 2 lists the used materials and corresponding colors as used in the figures within this section.

### 3.1. Trench Etching

**Figure 3:**
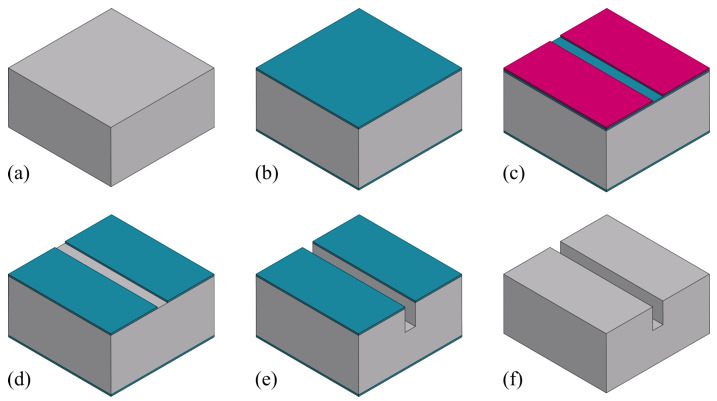
Fabrication steps to etch the trench structure: (**a**) Wafer inspection. (**b**) Wet thermal oxidation. (**c**) Photoresist spin-coating, exposure, and development. (**d**) RIE of t-SiO_2_ layer. (**e**) DRIE of silicon to obtain trench structures. (**f**) Removal of the t-SiO_2_ by HF.

Figure 3 illustrates the fabrication steps to etch trenches in the silicon substrate. First, a double-side polished wafer is inspected and the thickness is measured (Figure 3a). The wafer is oxidized by wet thermal oxidation to form a 1 μm thick t-SiO_2_ layer on the surface (Figure 3b). This t-SiO_2_ serves as a hard mask for the later trench etching step with deep reactive ion etching (DRIE) using a Bosch-based process. A photoresist is spin-coated, exposed, and developed (Figure 3c), and the pattern is transferred into the t-SiO_2_ layer by directional reactive ion etching (RIE, Figure 3d) using an O_2_/CHF_3_-based plasma described in Table 3. The designed line pattern is 50 μm wide, based on the study and analysis (see Section 2), and the desired depth of etched trenches is at least 150 μm, as the goal is to have a channel with a cross-section of 300 μm. The trenches are etched into the silicon by a two-step Bosch-based DRIE approach utilizing the cyclic SF_6_ and C_4_F_8_ plasma described in Table 4. After trench etching (Figure 3e), the wafer is cleaned with an in situ O_2_ plasma according to Veltkamp et al. [24] to strip fluorocarbon residue from the Bosch-based DRIE step. The t-SiO_2_ is then removed by a 50% hydrofluoric acid (HF) solution (Figure 3f).

**Figure 4:**
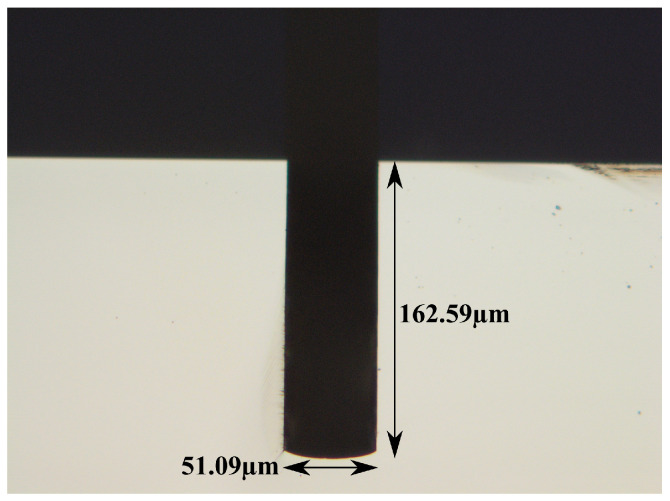
Cross-sectional optical microscope image of the etched trench.

Figure 4 shows a cross-sectional optical microscope image of an etched trench, and the measured depth is approximately 160 μm.

### 3.2. Trench Coating and Bottom Removal

**Figure 5:**
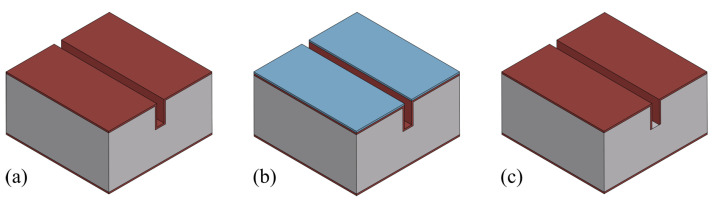
Fabrication steps to open trench bottom: (**a**) LPCVD of SiRN. (**b**) PECVD of SiO_2_. (**c**) Bottom coating layer removal by directional RIE.

**Figure 6:**
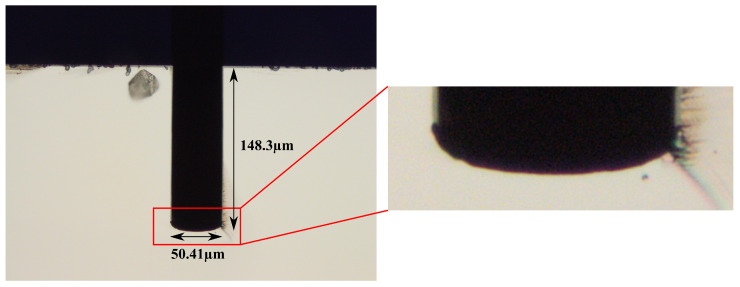
Cross-sectional optical microscope image and a closer look at the trench with an opened bottom. The small pieces close to the surface of the substrate are due to cleaving.

Figure 5 illustrates the fabrication steps to coat the substrate and open the trench bottom. First, a 1 μm thick SiRN layer is deposited by LPCVD (Figure 5a) according to the recipe in Table 5. This layer serves as a hard mask for the later channel etching by HNA solution. The opening of the trench bottom is performed by first depositing a 2 μm thick SiO_2_ layer by a PECVD method (Figure 5b) described in Table 6, followed by a directional RIE step (Figure 5c). The chosen layer thicknesses are based on the experience from previous studies and experiments [17]. The PECVD SiO_2_ will not reach the trench bottom due to the depth of the trench and the high process pressure of the PECVD step. Therefore, in the following etching step, the SiRN coating layer at the trench bottom is etched using the inductively coupled plasma RIE (ICP-RIE) recipe in Table 7, while the surface layer is protected. The result is shown in Figure 6.

### 3.3. Channel Etching

**Figure 7:**
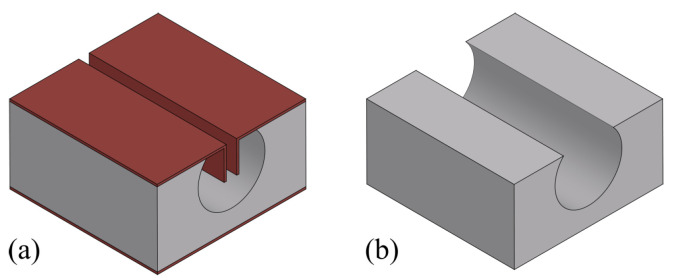
Fabrication steps to form the buried channels: (**a**) Channel etching by HNA solution. (**b**) Removal of SiRN hard mask by hot H_3_PO_4_.

**Figure 8:**
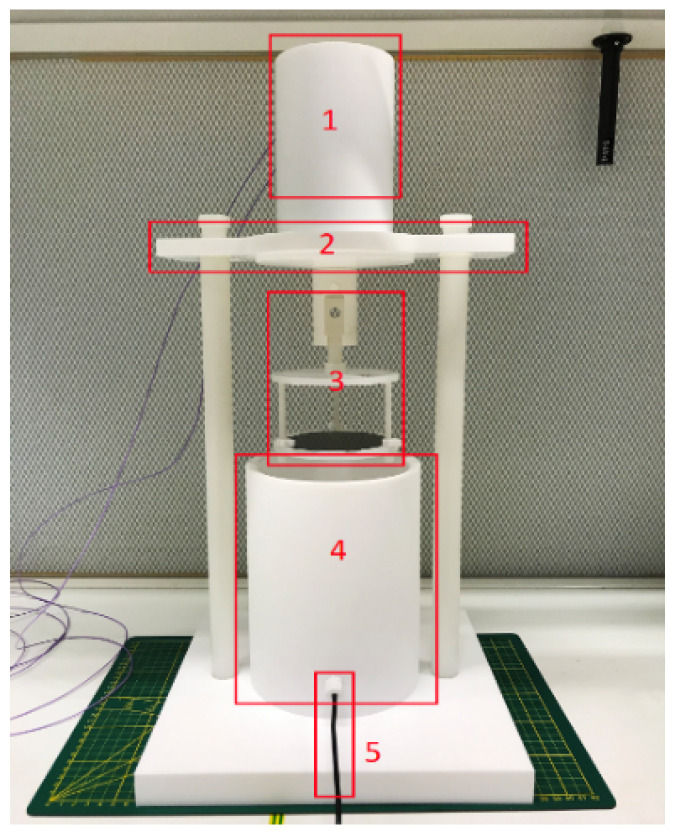
Dedicated reaction vessel for HNA etching: (1) Rotation motor. (2) Lid. (3) Wafer carrier. (4) Container. (5) Thermocouple.

After trench bottom removal, the channel can be formed. The fabrication steps are illustrated in Figure 7. The HNA solution is freshly prepared in a dedicated vessel [25] for this process (see Figure 8). The used composition here has a volumetric ratio of 50% HF solution, 69% HNO_3_ solution, and water in a ratio of 2:4:4. This composition can achieve a relatively high etch-rate and uniformity [25]. The wafer is mounted to the carrier and then submerged into the solution and etched for 2 h under room temperature. Wafer rotation is added with a speed of 10 rpm to improve the etching uniformity [26,27]. Finally, the SiRN masking layer is removed by hot phosphoric acid (H_3_PO_4_).

**Figure 9:**
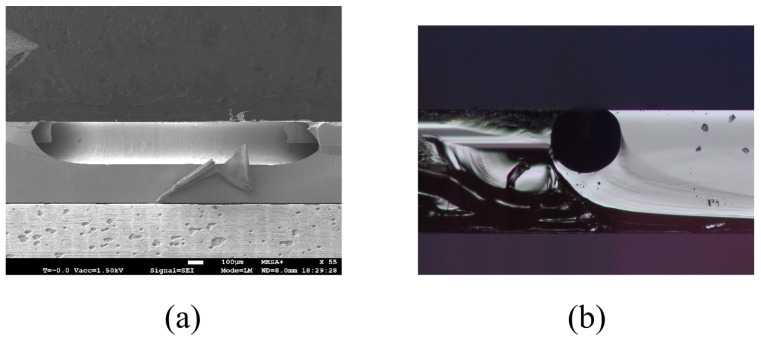
SEM and optical microscope images of etched channels: (**a**) Longitudinal-section of the etched channel. (**b**) Cross-section of one etched channel.

The result of etched channel is shown in Figure 9. By cutting the sample along the channel direction, the inner surface of channel can be seen clearly with SEM. Figure 9a shows the result of an etched channel. Figure 9b shows a cross-sectional optical microscope image. The cross-section is circular but with a flat top due to over-etching in the HNA solution. The measured width and depth are approximately 300 μm and 270 μm, respectively, and the width of the flat top is 190 μm.

### 3.4. Inlet/Outlet Etching

**Figure 10:**
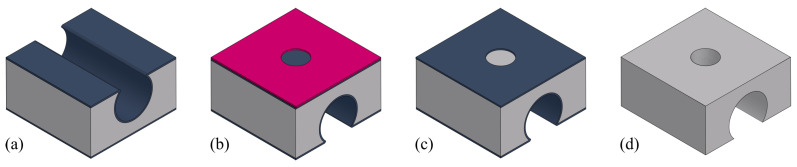
Fabrication steps to obtain inlet/outlet: (**a**) LPCVD of TEOS-based SiO_2_. (**b**) Photoresist spin-coating, exposure, and development. (**c**) RIE of TEOS-based SiO_2_. (**d**) Bosch-based DRIE of silicon to obtain inlets/outlets, and the removal of the TEOS-based SiO_2_ by 50% HF.

Once the channel is formed, inlets/outlets are to be etched from the backside of the wafer. Figure 10 illustrates the fabrication steps. First, a 1 μm layer of TEOS-based SiO_2_ is deposited by LPCVD (Figure 10a). This layer serves as a hard mask, as well as an etch-stop, for later inlets/outlets etching into silicon. The thickness of this layer should be as thick as possible since the silicon wafer needs to be etched through. The photoresist is spin-coated (Figure 10b), exposed with backside alignment to align the inlets/outlets pattern to the structures on the frontside, and then developed. The photoresist used here is a thicker one than in previous steps to ensure a full pattern transfer into the TEOS-based SiO_2_ layer. Bosch-based DRIE is used to etch inlet/outlet openings down to the TEOS-based SiO_2_ channel wall (Figure 10c). The depth to be etched in this step is approximately 250 μm. Inspection is performed by illuminating the backside of the wafer. If the light is visible from the front through the inlets/outlets apertures, it means the etching reached the channel wall. If not, the etching can be continued. The wafer is then cleaned with an O_2_ plasma to remove fluorocarbon residues and remaining photoresist. At the end, the TEOS-based SiO_2_ layer is stripped by 50% HF, so the channel is connected to the inlets/outlets (Figure 10d).

### 3.5. Thin-Film Bonding

**Figure 11:**
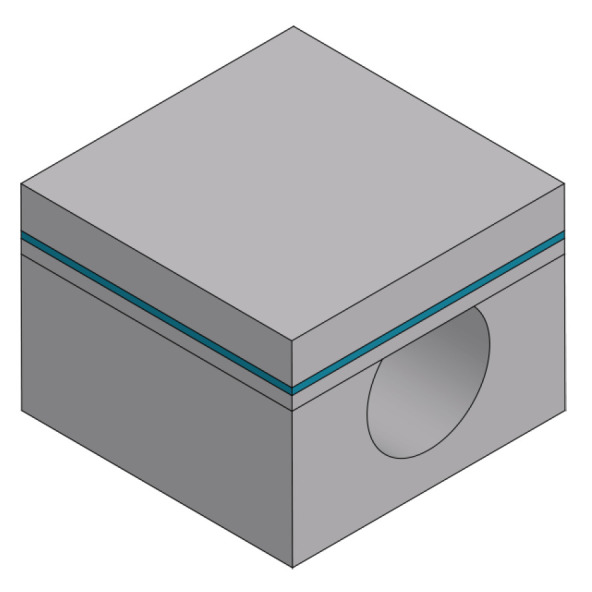
Device wafer and SOI wafer bonding.

Figure 11 illustrates the fabrication step. Wafer bonding can be performed in various ways with different materials. As a well-developed technique, many papers describe different bonding methods and materials [28]. In this work, only a thin-film layer is desired to be bonded on the device wafer. Therefore, the process is dived into two steps: wafer bonding and thin-film releasing (Section 3.6).

**Figure 12:**
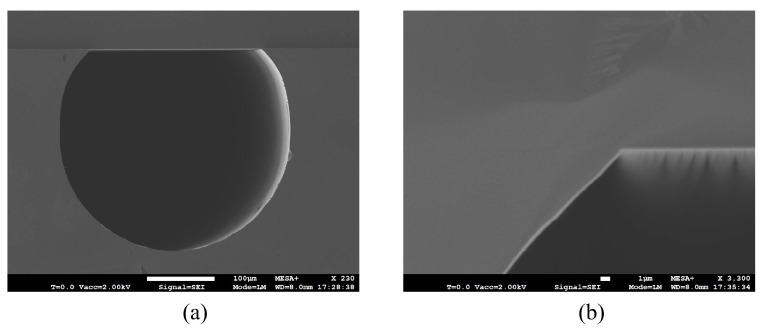
SEM images of wafer bonding result: (**a**) Channel cross-section after bonding. (**b**) A close-up of the interface showing a successful bonding as no obvious gap is observed.

Si–Si direct bonding is used as it is directly available in the MESA^+^ NanoLab facility. It is chosen not only because of its ease but also for the releasing step. A silicon on insulator (SOI) wafer is bonded to the channel wafer by first surface activation and prebonding, and then annealing at a high temperature (1050 °C). The bonded SOI wafer has a 500 μm thick handle layer, a 500 nm buried oxide layer, and a 10 μm thick device layer. The result is shown in Figure 12.

### 3.6. SOI Wafer Handle Layer Etching

**Figure 13:**
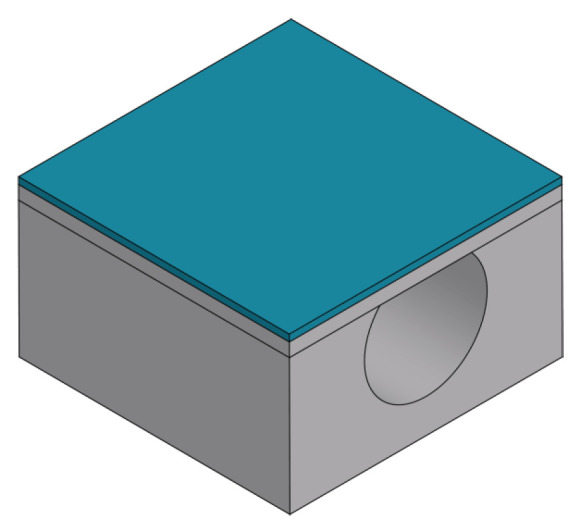
SOI wafer handle layer etching using SF_6_-based ICP-RIE.

After successfully bonding the channel wafer and SOI wafer, the handle layer of the SOI wafer must be removed. Figure 13 illustrates the fabrication steps. This is performed by using an ICP-RIE step with sulphur hexafluoride (SF_6_). In this process, the silicon loading is 100% and the volume of silicon to be etched is large, therefore, an ICP-RIE system with a high ICP power is used to maintain a certain etch rate. During the plasma etching process, the wafer is heated up by the exothermic behavior of the etch reaction. Therefore, the helium backside cooling system within the etcher is used for heat dissipation. So the SOI wafer on top is heated up by the plasma while the channel wafer beneath is cooled by helium. This will result in a difference in thermal expansion. Therefore, the recipe used here is designed to have cycles of etching and a waiting time to prevent large temperature difference. The wafer is also etched laterally 500 μm approximately from the edge, but it does not affect the channel structure because the extent of silicon on the sides of the channel is huge.

### 3.7. Channel Wall Formation

**Figure 14:**
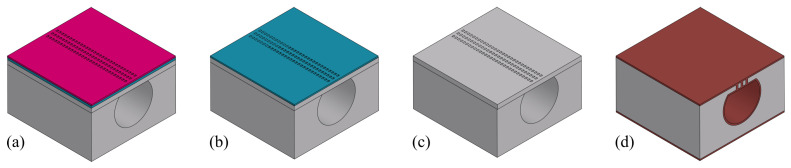
Fabrication steps to etch a slit pattern needed to grow the channel wall: (**a**) Photoresist spin-coating, exposure, and development. (**b**) RIE of t-SiO_2_ layer. (**c**) DRIE of silicon to obtain slits openings to the channel. (**d**) LPCVD of SiRN.

**Figure 15:**
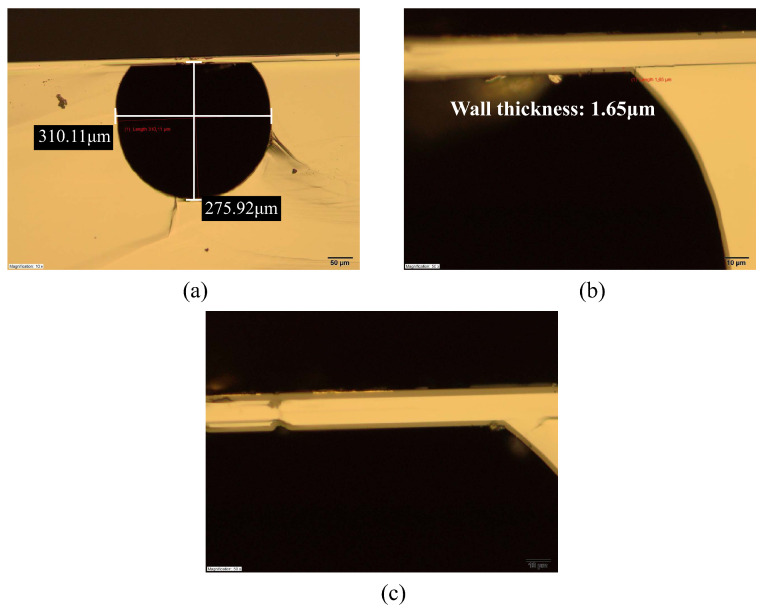
Optical microscope images of channel wall formation results: (**a**) Cross-section view of the channel. (**b**) Closer look at a bonding interface with defects, a thin SiRN channel wall is observed and the channel is closed successfully. (**c**) Closer look at another bonding interface without defects; a thin SiRN channel wall is observed, and the channel is closed.

After removing the handle layer of the SOI wafer, the channel wall can be formed. Figure 14 illustrates the fabrication steps to form the channel wall. The photoresist is spin-coated on the wafer. It is then exposed with a pattern containing arrays of slits that are 3 μm wide and 10 μm in length and developed (Figure 14a). The pattern is transferred into the SiO_2_ layer of the SOI wafer by RIE (Figure 14b). This SiO_2_ layer serves as a hard mask to etch the 10 μm silicon device layer of the SOI wafer, which is underneath. A 50% HF solution is then used to strip the SiO_2_ layer (Figure 14c). To close the channel, a 1.6 μm thick layer of SiRN is deposited by LPCVD (Figure 14d). The result is shown in Figure 15. It can be seen that even with an imperfect bonding, SiRN is still deposited and closing the channel (Figure 15b).

### 3.8. Integration of Actuation/Readout System

**Figure 16:**
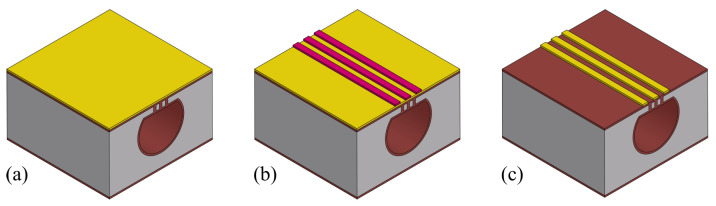
Fabrication steps to create electrodes for actuation/readout system: (**a**) Magnetron sputtering of the metal stack. (**b**) Photoresist spin-coating, exposure, and development. (**c**) Pattern transfer into metal layer by IBE and photoresist stripping.

After the channel wall formation step, electrodes for actuation and readout are fabricated. Figure 16 illustrates the fabrication steps to create the actuation/readout system. It starts with metal sputtering. A stack of 10 nm tantalum (Ta), 20 nm platinum (Pt), and 200 nm gold (Au) (from bottom to top) is sputtered by magnetron sputtering on the substrate consecutively without breaking the vacuum (Figure 16a). The photoresist is spin-coated (Figure 16b), exposed, and developed. Ion beam etching (IBE) using argon is used to pattern the metal layer stack. Secondary ion mass spectrometry (SIMS) is used to monitor the process and for end-point detection. After that, the remaining photoresist is stripped using an O_2_ plasma (Figure 16c).

### 3.9. Channel Release Etching

**Figure 17:**
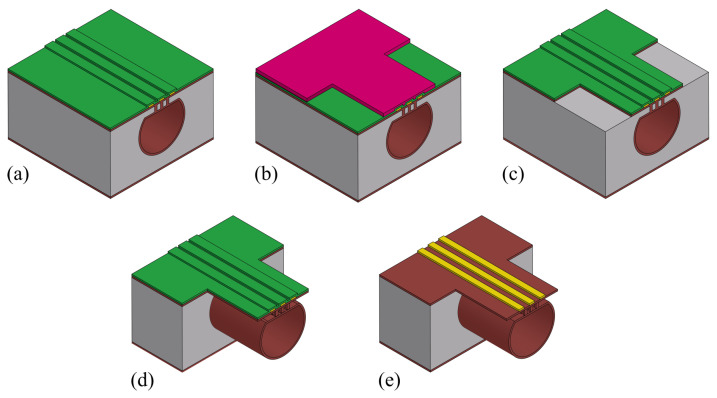
Fabrication steps to release the channel: (**a**) Evaporation of AlO_x_. (**b**) Photoresist spin-coating, exposure, and development. (**c**) RIE of AlO_x_ and SiRN layers. (**d**) Bulk silicon etching using SF_6_-based ICP-RIE to release the channel. (**e**) Removal of AlO_x_ by RIE to expose the electrodes for electrical connection.

The final step of the process is to release the microfluidic channels from the substrate to allow for vibration in the envisioned application. This is achieved by etching away the bulk silicon around the channel. Figure 17 illustrates the fabrication steps. Aluminum oxide (AlO_x_) is used as hard mask material due to its high selectivity. The aluminum oxide layer is deposited by electron beam evaporation and covers the metal traces on the wafer (Figure 17a). The photoresist is then spin-coated (Figure 17b), exposed, and developed. An ICP-RIE step with a boron trichloride (BCl_3_) and hydrogen bromide (HBr) plasma is used to pattern the AlO_x_. The SiRN layer underneath is then etched through (Figure 17c).

Because the microfluidic channel structure has a large cross-sectional area, deep trenches are first etched beside the channels to accelerate the release etching step. After that, SF_6_ plasma is used for bulk silicon etching. Again, this is performed by alternating etching and waiting to prevent burning the channel (Figure 17d). At the end, the remaining AlO_x_ layer is stripped to reveal the electrode (Figure 17e).

**Figure 18:**
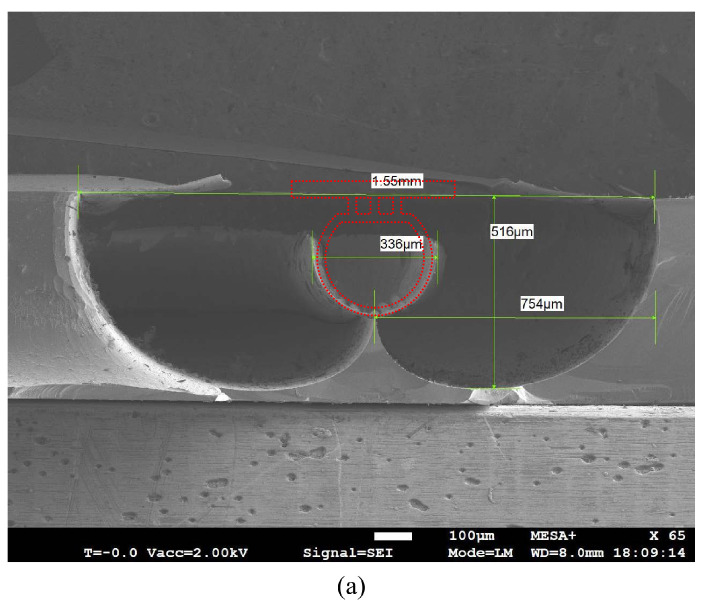

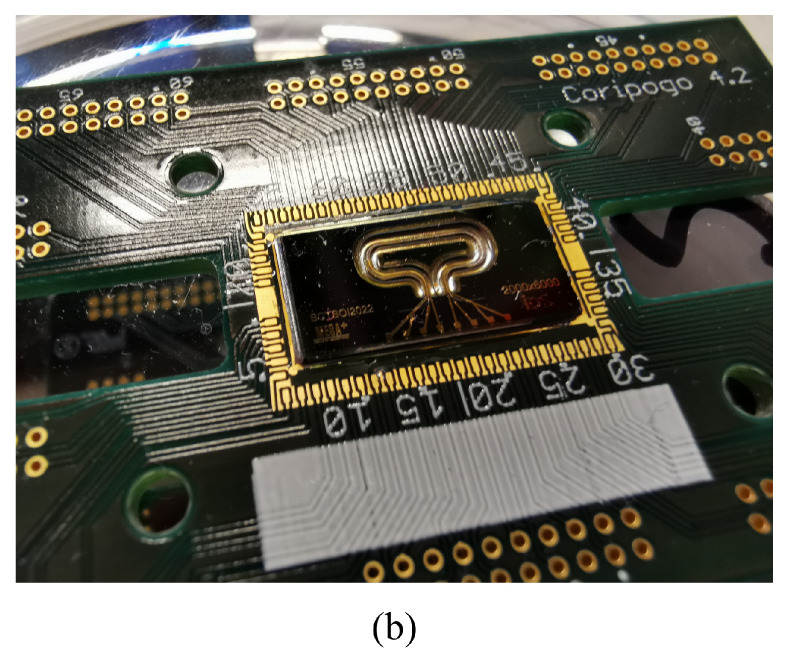
Pictures of fabrication results: (**a**) Cross-sectional SEM image of the channel with illustrated contour of the channel. (**b**) Photograph of a fabricated demonstrator device after mounting it on a dedicated PCB.

Figure 18a shows a SEM image of the fabricated microfluidic channel after the release etching step. From the SEM image, it can be seen that the silicon substrate is almost etched through but the channel is still not entirely released from the substrate. A small part at the bottom still attaches the channel to the silicon substrate and a slightly longer etching time would have been needed for a full release. The channel is more than 300 μm wide and has a channel wall thickness of only 1.5 μm. Therefore, it is extremely difficult to cleave/dice/cut the channel and observe a cross-section without breaking it. Figure 18b shows a photograph of the structure of a complete micro-Coriolis mass flow sensor that was realized to demonstrate the capabilities of the process.

## 4. Discussion

This fabrication run is the very first one to demonstrate the idea of forming a large channel based on BCT with a wafer bonding step. The preliminary results show the feasibility and potential. However, further modification and optimization are needed. The key step, wafer bonding, still has many options.

**Figure 19:**
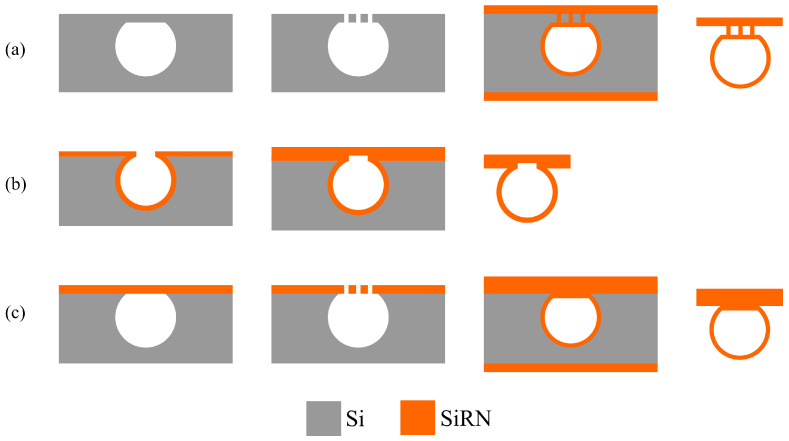
Bonding with different materials and the resulting channel cross-section after the release etching: (**a**) Si–Si bonding (presented in this work). (**b**) SiRN–SiRN bonding. (**c**) Si–SiRN bonding.

For the wafer bonding step, depending on the material of the thin film to be bonded, the further steps in the fabrication process can vary. Considering the further fabrication steps (channel wall formation, release etching, etc.), a SiRN thin film is optimal. Figure 19 illustrates different situations. One way is that another SiRN layer is deposited on the channel wafer, and then bonded with a silicon wafer with SiRN layer (Figure 19b). This results in a sealed channel with a chemically inert inner surface. No more extra channel wall formation steps are required. However, this requires a uniform SiRN–SiRN bonding with good quality since any defects can lead to leakage in the device. The other way is that the SiRN surface is directly bonded to the channel wafer, i.e., no LPCVD of SiRN on the channel wafer (Figure 19c). The channel will then be similar to SCT channels [29] and the further fabrication steps can be designed easily. After removing the substrate (handle layer), the bonded SiRN layer can be patterned and the apertures can be etched through. Followed by an LPCVD step, the channel wall can be formed. However, there are two main issues: one is the difficulty of bonding SiRN surfaces due to their chemical inertness; the other is the release of the substrate. Reck et al. published a paper about bonding silicon nitride surfaces in 2011 [30]. In this work, two silicon nitride surfaces are successfully bonded. According to this study, bonding silicon nitride surface is difficult as it requires extremely smooth surfaces (roughnesses of only a few nanometres are allowed), and a layer thickness of no more than 440 nm is possible. Moreover, to release the bonded thin film, the silicon substrate needs to be etched away completely. This can be performed using an SF_6_ plasma. However, it also etches SiRN and, therefore, excludes this material as a possible etch-stop material.

In our fabrication process, bonding Si–Si surfaces is the easiest, but the consequence is not optimal. In the release etching step (Figure 17d), the bonded silicon layer (device layer of the SOI wafer) is also etched from the side. It then results in a hollow structure between two SiRN layers with only pillars connecting them. This part can then be fragile and affect subsequent processing. Moreover, the deposited SiRN channel wall layer is only 1.5 to 1.6 μm thick due to the layer material property and the limitation of the machine, but the top thin film is more than 100 μm wide due to the design. This free-hanging part can be damaged easily. In several chips, thin films are seen partly detached from the channel while still attaching to the silicon. Due to the internal stress, these thin films bend upwards or break into pieces. If SiRN is used for bonding instead of silicon, these problems can be prevented. The final result will be a channel similar to SCT channels, with a thicker (than bonding silicon thin film) flat top and no pillar structures, which should increase the durability and robustness.

The optimization of designing the electrical system (electrode, metal track, etc.) can also be helpful, as well as the channel features like the dimension of the flat top or the apertures for channel wall formation, to increase mechanical strength. Different actuation and readout methods can be integrated just like in SCT [31,32,33,34,35].

The inspection/monitoring of the process needs to be optimized as well or new methods should be used, especially after the formation of channels. As mentioned earlier, it becomes much more difficult to cut the substrate with such large hollow structures.

## 5. Conclusions

In this work, a fabrication process is presented for the realization of large free-hanging microfluidic channels with a thin channel wall. As a demonstration, a microfluidic channel with a cross-section of more than 300 μm in diameter and a channel wall of only 1.5 μm thick is fabricated. The obtained channel is fully integrated into a single silicon substrate. Thanks to the high selectivity of the HNA etchant between silicon and SiRN, the maximum diameter of the channel is mainly determined by the depth of a pre-etched trench. Therefore, channels with an even larger dimension can be achieved by this method, especially when thicker wafers are used. Some details and discussions are presented to point out the direction of further modification and optimization of the process. Future work will focus on using this technology to fabricate a fully operational high-flow micro-Coriolis mass-flow and density sensor, as well as other microfluidic devices.

## Figures and Tables

**Table 1:** Effect of parameters on the fabrication steps, arrows mean the increase/decrease in the value of parameters, −/+/O symbols mean the negative/positive/neutral effect on the results and ease of processes.

Parameter	Trend	Bottom Removal	Channel Etch	Channel Dimension
$h_{t}$	↑	−	−	+
	↓	+	+	−
$w_{t}$	↑	+	+	O
	↓	−	−	O
$t_{m1}$	↑	−	+	+
	↓	+	−	−
$t_{m2}$	↑	+	O	O
	↓	−	O	O

**Table 2:** Legend for the materials used in the fabrication process.

Material	Name and Abbreviation
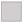	Silicon (Si)
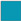	Thermal silicon dioxide (t-SiO_2_)
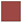	Silicon-rich silicon nitride (SiRN)
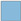	PECVD SiO_2_ (PECVD SiO_2_)
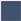	LPCVD tetraethyl orthosilicate-based SiO_2_ (TEOS)
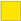	Metal stack of Ta, Pt, and Au (Metal)
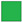	E-beam evaporated aluminum oxide (AlO_x_)
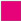	Photoresist (PR)

**Table 3:** Settings for directional RIE of SiO_2_ in a Plasma-Therm 790 (Plasma-Therm, LLC, Saint Petersburg, Florida, United States of America).

Setting	Unit	Value
Temperature	[°C]	10
CHF_3_ flow	[SCCM]	100
O_2_ flow	[SCCM]	5
Process pressure	[mTorr]	40
Power	[W]	250
Electrode	[-]	Graphite

**Table 4:** Settings for Bosch-based etching of HAR trenches in an Oxford Instruments PlasmaPro100 Estrelas (Oxford Instruments plc, Bristol, UK).

Setting	Unit	Deposition	Etching
Temperature	[°C]	25	25
He BSC pressure	[Torr]	10	10
Time	[s]	2.4	3.0
ICP	[W]	1300	1600
${\mathrm{CCP}}_{\mathrm{HF}}$	[W]	5	0
${\mathrm{CCP}}_{\mathrm{LF}}$	[W]	0	16
Pressure	[mTorr]	25	40
C_4_H_8_ flow	[SCCM]	200	10
SF_6_ flow	[SCCM]	10	200

**Table 5:** Recipe used for the LPCVD of low-stress (50 MPa) SiRN in a TS6304 furnace of Amtech Tempress Systems (Amtech Tempress Systems BV, Vaassen, The Netherlands).

Setting	Unit	Value
Temperature	[°C]	850
SiH_2_Cl_2_ flow	[SCCM]	72
NH_3_ flow	[SCCM]	22
N_2_ flow	[SCCM]	150
Process pressure	[mTorr]	150

**Table 6:** Recipe used for the PECVD of SiO_2_ in an Oxford Instruments PlasmaLab80 (Oxford Instruments plc, Bristol, UK).

Setting	Unit	Value
Temperature	[°C]	300
2% SiH_4_ in N_2_ flow	[SCCM]	200
N_2_O flow	[SCCM]	710
Process pressure	[mTorr]	650
LF power	[W]	60

**Table 7:** Settings for the ICP-RIE of the bottom LPCVD SiRN layer in an Oxford Instruments PlasmaPro100 Cobra (Oxford Instruments plc, Bristol, UK).

Setting	Unit	Value
Temperature	[°C]	10
He BSC pressure	[Torr]	10
ICP power	[W]	1750
${\mathrm{CCP}}_{\mathrm{RF}}$ power	[W]	50
SF_6_ flow	[SCCM]	50
O_2_ flow	[SCCM]	20
Process pressure	[mTorr]	20

## Data Availability

The original contributions presented in the study are included in the article, further inquiries can be directed to the corresponding author.
